# Structural characterization of a protein adsorbed on aluminum hydroxide adjuvant in vaccine formulation

**DOI:** 10.1038/s41541-019-0115-7

**Published:** 2019-05-28

**Authors:** Linda Cerofolini, Stefano Giuntini, Enrico Ravera, Claudio Luchinat, Francesco Berti, Marco Fragai

**Affiliations:** 1 Magnetic Resonance Center (CERM), University of Florence and Consorzio Interuniversitario Risonanze Magnetiche di Metallo Proteine (CIRMMP), Via L. Sacconi 6, 50019 Sesto Fiorentino, Italy; 2 Department of Chemistry, University of Florence, Via della Lastruccia 3, 50019 Sesto Fiorentino, Italy; 3 Technical R&D, GSK Vaccines, Via Fiorentina 1, 53100 Siena, Italy

**Keywords:** Biologics, Vaccines

## Abstract

The heterogeneous composition of vaccine formulations and the relatively low concentration make the characterization of the protein antigens extremely challenging. Aluminum-containing adjuvants have been used to enhance the immune response of several antigens over the last 90 years and still remain the most commonly used. Here, we show that solid-state NMR and isotope labeling methods can be used to characterize the structural features of the protein antigen component of vaccines and to investigate the preservation of the folding state of proteins adsorbed on Alum hydroxide matrix, providing the way to identify the regions of the protein that are mainly affected by the presence of the inorganic matrix. l-Asparaginase from *E. coli* has been used as a pilot model of protein antigen. This methodology can find application in several steps of the vaccine development pipeline, from the antigen optimization, through the design of vaccine formulation, up to stability studies and manufacturing process.

## Quality control: Assessing how adjuvants affect vaccine antigens

Aluminum salts are the most frequently used adjuvants in vaccines, but they may negatively affect the antigens in suspension via chemical interactions that could reduce antigen quality and stability. Marco Fragai from the University of Florence and colleagues now report a method based on solid-state nuclear magnetic resonance, to characterize the structure of the model antigen *L*-asparaginase when adsorbed to the adjuvant aluminum hydroxide in a vaccine suspension. The researchers identify the antigen motifs that interact with the adjuvant directly, and are able to shed light on the antigen’s folding when in complex with the aluminum adjuvant in suspension. This approach could help identify antigen misfolding as a consequence of interactions with chemical adjuvants and may provide useful information during multiple stages of vaccine manufacturing, from antigen optimization to the vaccine manufacturing process, to ultimately improve vaccine quality.

## Introduction

Vaccination is one of the major contributors to the control of infections in human population globally. Insoluble aluminum salts have been used to enhance the immunogenicity of antigens against bacterial and viral infections since 1926, when Glenny et al. reported their use to improve the response of diphteria toxoid.^1^ Recently other adjuvants based on oil-in-water emulsions (i.e. MF59, AS03) and liposomes have been used for licensed vaccines and other candidates at different stages of research and development.^2,3^ However, the aluminum salts are the most commonly used adjuvants for commercial vaccines and, also due to their long-term success, they still remain the “gold standard” against a new adjuvant.^4^

For licensed vaccines, aluminum(III) hydroxide (AlumOH) and aluminum(III) phosphate (AlumP) are the most commonly used adjuvants. AlumOH is a chemically crystalline aluminum(III) oxyhydroxide (AlOOH), prepared by exposing soluble aluminum(III) salts (generally Al(H_2_O)_6_Cl_3_ or AlK(SO_4_)_2_) to alkaline conditions to obtain a suspension which is finally dehydrated under hydrothermal conditions. AlumP is a noncrystalline compound generated by incorporation of phosphate which interferes with the crystallization process. AlumP can be prepared by mixing the aluminum salt Al(H_2_O)_6_Cl_3_ or AlK(SO_4_)_2_ with a basic solution of trisodium phosphate, or directly mixing aluminum salt with phosphate solution, followed by precipitation with sodium hydroxide. The substitution of hydroxyl groups of AlumOH with phosphate groups results in the formation of aluminum hydroxyphosphate, Al(OH)_x_(PO_4_)_y_, a nonstoichiometric compound in which the ratio of hydroxyls to phosphate depends on the precipitation conditions.^4^

In the final formulation the antigen is adsorbed on the Alum-based adjuvant and administered as precipitate. After the injection, a fraction of the antigen is released in the extracellular fluid and cleared from the injection site. These adjuvants enhance the immune response by a slow release of the antigen from the injection site and, more important, through activation of the dendritic cells and stimulation of CD4+ T cells.^5^ However, probably due to the complexity and several immunological pathways operating simultaneously, the mechanism of action of aluminum adjuvants for enhancing the immune response remains not fully understood, although they have been used over many years in vaccines for human use.

Electrostatic interaction, phosphate ligand exchange, hydrogen bonding and van der Waals interactions may be involved in the adsorption mechanism depending on antigen, adjuvant, excipients, pH and ionic strength.^6^ However, the interaction with the adjuvant may alter folding, conformation and stability of the antigen.^6^ For folded protein antigens, electrostatic forces are reported to dominate the interaction with the AlumOH hydrated gel in a manner dependent on the pH and isoelectric point.^7^ Alteration of folding and native conformational state of the epitopes may affect the immune response by influencing the antigen processing and presentation, the amount of protein bound to the adjuvant and its binding affinity.^8^ Also the long-term stability of the antigen in the final formulation is extremely important for the effectiveness and commercial viability of the vaccine. Therefore, the characterization of the protein antigen bound to the aluminum gel adjuvant is particularly relevant for the development of more effective and stable vaccine. Unfortunately, the heterogeneous composition of vaccines has hampered for long time the biophysical and structural characterization of the protein component within the formulation. Desorption from the aluminum adjuvant and elution have often been used to analyze post-formulation antigens. However, this strategy does not provide structural information on the antigen when adsorbed on the adjuvant. Attenuated total reflectance Fourier-transformed infrared and fluorescence spectroscopy, circular dichroism and differential scanning calorimetry have been used to investigate adjuvant-interacting protein antigens.^9,10^ More recently, electron microscopy has been used to characterize the antigens in adjuvant bound states.^11^ However, this approach is not for general use, and it may be feasible only with extremely large proteins or protein assemblies.

In the last years, solid-state NMR is emerging as an outstanding spectroscopic technique in structural biology,^12–25^ and to investigate, at atomic detail, difficult protein systems in many different states, including biosilica-entrapped proteins, hydroxyapatite-protein composites, PEGylated and polysaccharide-conjugated proteins and proteins grafted onto nanoparticles.^26–33^ In particular, soluble proteins and protein assemblies,^34^ membrane proteins, such as bacterial porins^17^ and transmembrane helix proteins,^35^ viral capsid components^36^ and RNA^37^ have been characterized by solid-state NMR spectroscopy, opening up promising opportunities for the study of different classes of antigens. Here we show that atomic structural details on protein antigens adsorbed on Aluminum gel adjuvant can be achieved by solid-state NMR from vaccine formulations obtained starting from isotopically enriched antigens and stored for several months. The following NMR analysis performed on *Escherichia coli* l-Asparaginase (ANSII) sheds light (i) on the folding state of the protein bound to aluminum adjuvant, (ii) on the protein regions involved in the interaction with gel, and (iii) it provides a new tool for vaccines formulation development and stability studies.

## Results

### NMR spectroscopy

**Fig. 1:**
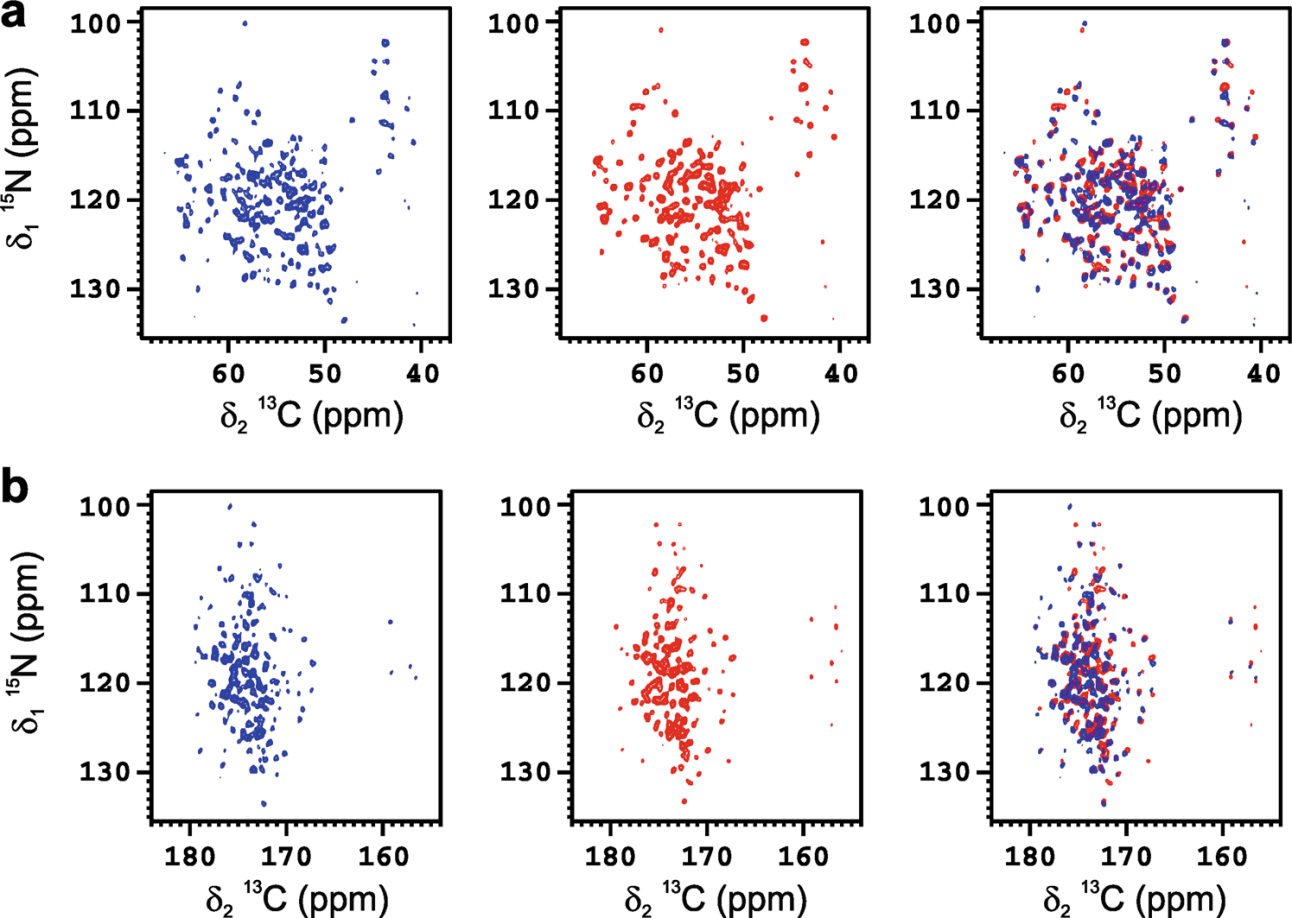
2D ^15^N ^13^C NCA **a** and NCO **b** spectra of ANSII-AlumOH (blue, left) and rehydrated freeze-dried ANSII (red, middle); a superposition of the two NCA and NCO spectra, respectively, is also displayed to help in the comparison (right). The spectra were acquired at ~290 K, MAS 14 kHz and 800 MHz

AlumOH adjuvanted formulation of uniformly isotopically enriched ANSII [U-^13^C-^15^N] was used as vaccine model and investigated by solid-state NMR. Despite the low amount of protein absorbed on the inorganic matrix, the 2D amide-carbon alpha (2D ^15^N ^13^C NCA) and amide-carbonyl (2D ^15^N ^13^C NCO) correlation spectra of ANSII-AlumOH (Fig. 1a, b, respectively), collected on sedimented material obtained by centrifugation, are of high quality, and comparable for the number of cross-peaks detected to the 2D ^15^N ^13^C NCA and 2D ^15^N ^13^C NCO spectra collected on rehydrated freeze-dried ANSII. In both N−C correlation spectra the resonances are superimposable to those of the rehydrated freeze-dried ANSII (Fig. 1), immediately demonstrating that the three-dimensional structure of the protein is preserved after adsorption on the inorganic salt. The assignment of the 2D ^15^N ^13^C NCA spectrum of ANSII-AlumOH (see Supplementary Table 1) was easily obtained by comparison with the 2D ^15^N ^13^C NCA collected on the rehydrated freeze-dried ANSII, and also using the information from the 2D ^13^C-^13^C correlation spectrum (dipolar assisted rotational resonance, DARR) acquired on ANSII-AlumOH.

### Data analysis

**Fig. 2:**
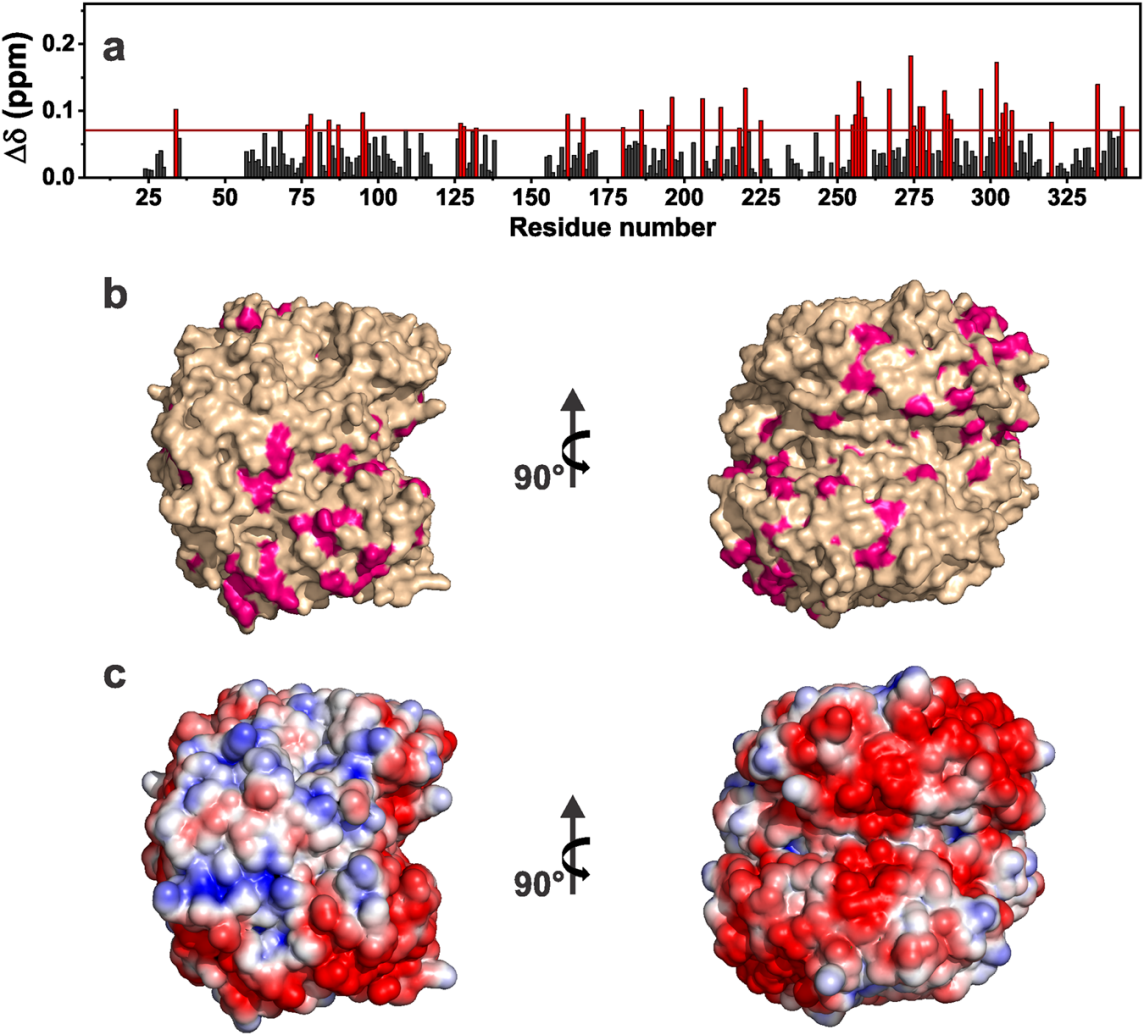
Chemical shift perturbations (CSP) of ANSII-AlumOH with respect to rehydrated freeze-dried ANSII, evaluated according to the formula ${\mathrm{\Delta }}\delta = \frac{1}{2}\sqrt {(\Delta \delta _{C\alpha }/2)^2 + (\Delta \delta _N/5)^2}$.^53^ The residues experiencing variations larger than the standard deviation (red dashed line) have been highlighted in red **a**. CSP mapping on the protein surface (PDB code: 3ECA) with the region with the largest perturbation in magenta **b**. Electrostatic potential generated by APBS plugin in PyMOL on 3ECA with blue and red representing the regions of positive and negative electrostatic potential, respectively **c**

The assignment of the spin systems allowed the analysis of the chemical shift perturbation (CSP). The CSP data reveal that the residues experiencing the largest changes are located on the protein surface with negative electrostatic potential (Fig. 2). In particular, the largest CSP are observed for residues in the region between Ala-250 and Lys-310, which possesses a wide distribution of negative charge. The presence of electrostatic interactions between the negatively charged surface of the protein and the positive surface of the inorganic material is further supported by the properties of the aluminum oxyhydroxide that has an isoelectric point of about 11.4, and at pH 7.5 exhibits a positive surface charge.^38^ These findings are consistent with the mechanism proposed for the adsorption of folded antigen proteins onto the AlumOH.

## Discussion

The application of structural biology to develop new vaccines has already proved its effectiveness.^39^ Engineered antigens incorporating protective determinants have been developed by NMR-based structural methodologies to improve protection, safety and industrial scale production.^40^ Recently, we have demonstrated that high-resolution SSNMR, based on ^13^C detection, can be applied to assess the preservation of the folding of silica-encapsulated enzymes,^27^ and track the chemical shift perturbation on the protein surface induced by the interaction with ligands.^41^ ANSII from *E. coli* is a homotetrameric assembly of 138 kDa with D_**2**_ symmetry. This protein is in clinical use since 1967 against childhood acute lymphoblastic leukemia but has now been largely replaced by its PEGylated form, which exhibits longer‐lasting activity and, more important, a lower immunogenicity.^42^ The previously reported characterization of native ANSII and its antigenic properties make it a suitable model to investigate the potential of new NMR methods for vaccines development and characterization. The amount of protein antigen adsorbed to the adjuvant and the heterogeneity of the vaccine formulation are two important limiting factors for their biophysical characterization. However, these limitations can be overcome today by using the high sensitivity of solid-state NMR combined with isotopic labeling methodologies. Therefore, the use of solid-state NMR and structural methodologies to characterize adsorbed antigens promises to solve several challenges frequently encountered in vaccine development. For example, the destabilization of protein antigen structure upon the adsorption to the aluminum adjuvants has been suggested to play a role in immune stimulation. In particular, protein unfolding may favor the proteolytic degradation of the antigen and the presentation of the fragments to the immune cells.^6^ At the same time, for some vaccines, other studies have shown that the loss of the native secondary and tertiary structure can result in partial loss of immunogenicity.^43,44^ In this respect, the information provided by solid-state NMR on folding preservation could be decisive to determine the molecular basis of the loss of efficacy and to design vaccine with an improved immunogenicity. A further potential application of the solid-state NMR to vaccine development concerns the optimization of the experimental conditions for the adsorption of antigen to aluminum salts. Usually, the adsorption of the antigen protein is optimized by changing the pH and buffer components that directly affect the electrostatic interactions (i.e. zeta potential). However, the amount of protein antigen adsorbed to the aluminum salt is determined by measuring the concentration of the residual free protein in solution without any quantitative and qualitative information on the protein bound to the adjuvant. In this respect, the collection of mono- and multidimensional solid-state NMR spectra on formulations containing isotopically enriched protein antigens allows for a completely new approach providing semiquantitative information on the adsorbed protein antigen and on its folding state, suitable for driving structure-based optimization of vaccine formulation and manufacturing process. Also accelerated stability studies and mechanistic studies to investigate the exposure to high temperatures, freeze-thaw events and low pH would benefit from the use of this new methodology. Moreover, it should be pointed out that this methodology can be applied to a wide range of proteins because the labeling of antigens in eukaryotic expression systems, although highly expensive for academia, is nowadays feasible.^45–50^ Finally, the extension of the cryo-probe technology to solid-state NMR and the forthcoming increase in magnetic field strength of the NMR instruments are expected to improve further the sensitivity of the experiments allowing a more detailed characterization, or decreasing the amount of protein required for the detection.

## Methods

### Expression and purification of uniformly isotopically enriched ANSII [U-^13^C-^15^N]

*Escherichia coli* C41(DE3) cells were transformed with pET-21a(+) plasmid encoding ANSII gene. The cells were cultured in ^13^C, ^15^N-enriched minimal medium (M9) containing 0.1 mg/mL of ampicillin, grown at 310 K, until OD_600_ reached 0.6–0.8, then induced with 1 mM isopropyl *β*-d-1-thiogalactopyranoside. They were further grown at 310 K overnight and then harvested by centrifugation at 6500 rpm (JA-10 Beckman Coulter) for 15 min at 277 K. The pellet was suspended in 10 mM Tris-HCl, pH 8.0, 15 mM EDTA, 20% sucrose buffer (60 mL per liter of culture) and incubated at 277 K for 20 min upon magnetic stirring. The suspension was centrifuged at 10,000 rpm (F15-6x100y Thermo Scientific) for 30 min and the supernatant discarded. The recovered pellet was re-suspended in H_2_O milli-Q (60 mL per liter of culture) and newly incubated at 277 K for 20 min under magnetic stirring. Again the suspension was centrifuged at 10,000 rpm (F15-6x100y Thermo Scientific) for 30 min. The pellet was discarded, whereas the supernatant was treated with ammonium sulfate. Still under magnetic stirring, solid ammonium sulfate was added in aliquots up to 50% saturation. The precipitate was removed by centrifugation, then further ammonium sulfate was added up to 90% saturation to trigger the precipitation of ANSII, which was recovered again by centrifugation. The precipitated ANSII was re-dissolved in a minimal amount of 20 mM Tris-HCl buffer at pH 8.6 and dialyzed extensively against the same buffer. ANSII was purified by anionic-exchange chromatography using a HiPrep Q FF 16/10 column (GE Healthcare Life Science). The protein was eluted in 20 mM Tris-HCl buffer at pH 8.6 with a linear 0–1 M NaCl gradient. Fractions containing pure ANSII were identified by Coomassie staining SDS-PAGE gels, then joined and dialyzed extensively against 50 mM phosphate buffer at pH 7.5.

### Preparation of vaccine formulation

Commercially available AlumOH (Sigma) was used. To reduce the phosphate content and assure a complete adsorption to AlumOH adjuvant, 10 mL of ANSII [U-^13^C-^15^N] protein at the concentration of 0.583 mg/mL in 150 mM sodium phosphate (pH 7.5) was dialyzed and concentrated to 2 mL volume by using a Vivaspin 5 kDa molecular weight cutoff membrane (Sartorius). A protein concentration of 2.77 mg/mL was obtained as estimated by MicroBCA commercial kit (Thermo).

Fifty milliliters of AlumOH adjuvanted formulation at a protein concentration of 100 µg/mL (with 2 mg/mL of Al(OH)_3_ and 9 mg/mL of NaCl) was prepared by mixing 1.805 mL of ANSII [U-^13^C-^15^N] (5 mg totally), 23.195 mL MilliQ H_2_O, 25 mL AlumOH at 4 mg/mL Alum(OH)_3_ and 18 mg/mL NaCl. To estimate the amount of protein adsorbed to the AlumOH adjuvant, the hydrogel was pelleted at 15,000 rpm for 1 min and the protein content estimated on the supernatant.

### Sample preparation and NMR measurements

SSNMR experiments were recorded on a Bruker Avance III spectrometer operating at 800 MHz (19 T, 201.2 MHz ^13^C Larmor frequency) equipped with Bruker 3.2 mm Efree NCH probe-head. All spectra were recorded at 14 kHz MAS frequency and the sample temperature was kept at ~290 K.

The sample of ANSII-AlumOH was stored for 6 months at 277 K to reproduce a possible shortest shelf-life of a commercial vaccine. A mild centrifugation was used to separate the colloidal AlumOH adjuvant with the adsorbed ANSII from the supernatant. The hydrogel was pelleted at 10,000 rpm for 1 h at 4 °C using an ALC multispeed refrigerated PK121R centrifuge (rotor model A-M10). Then, the precipitate was used to fill a Bruker 3.2 mm thin-wall zirconia rotor. Silicon plugs (courtesy of Bruker Biospin) placed below the turbine cap were used to close the rotor and preserve hydration. The rotor was filled with 30 mg of wet precipitate.

A batch of freeze‐dried ANSII [U- ^13^C, ^15^N, ca. 20 mg of material] was prepared as reference sample. The protein material was packed into a Bruker 3.2 mm zirconia rotor, and rehydrated with a solution of 9 mg/mL NaCl in MilliQ H_2_O, to simulate the same conditions of ANSII-AlumOH. The hydration process was monitored through 1D {^1^H}-^13^C cross-polarization SSNMR spectrum and stopped when the resolution of the spectrum did not change any further for successive additions of the solution.^26,29,30^

The amount of protein present in the NMR sample of ANSII-AlumOH was estimated to be 0.7–1 mg (per 20–25 mg of AlumOH), according to the relative intensity of 1D {^1^H}-^13^C cross-polarization spectra recorded on ANSII-AlumOH and on the sample of rehydrated freeze-dried ANSII containing a known amount of protein.

Standard ^13^C-detected SSNMR spectra (2D ^15^N ^13^C NCA, 2D ^15^N ^13^C NCO and 2D ^13^C-^13^C DARR, mixing time 50 ms) were acquired using the pulse sequences reported in the literature.^51^ Pulses were 2.6 μs for ^1^H, 4 μs for ^13^C and 5.6 μs for ^15^N. The inter-scan delay was set to 1.5 s in all the experiments. The number of scans used for the acquisition of 2D ^15^N ^13^C NCA and ^15^N ^13^C NCO experiments was 4096 and 128 for ANSII-AlumOH and rehydrated freeze-dried ANSII, respectively. Each N−C correlation experiment collected on ANSII-AlumOH was acquired for 6 days while the 2D ^13^C-^13^C DARR (number of scans equal to 656) required 8 days of acquisition (additional experimental information is reported in Supplementary Table 2).

No significant protein desorption was observed to occur by spinning the sample at 14 kHz as proved by the comparison of the 1D {^1^H}-^13^C cross-polarization spectra collected just after sample preparation and after the whole NMR characterization: in the two 1D {^1^H}-^13^C cross-polarization spectra the signal intensity is approximately the same (Supplementary Fig. 1).

All the spectra were processed with the Bruker TopSpin 3.2 software package and analyzed with the program CARA.^52^

## Supplementary information

Supplemental Material

## Data Availability

All data generated or analyzed during this study are included in this published article and its supporting information file.

## Supplementary information

**Supplementary information** accompanies the paper on the *npj Vaccines* website (10.1038/s41541-019-0115-7).
