# Supplementary material for: Structural characterization of a protein adsorbed on aluminum hydroxide adjuvant in vaccine formulation
Source: NPJ Vaccines. 2019 May 28;4:20. doi: 10.1038/s41541-019-0115-7 (PMC6538755; doi:10.1038/s41541-019-0115-7)
Supplement: Supplementary file 1 — Supplemental Material [file 41541_2019_115_MOESM1_ESM.pdf]

Structural characterization of a protein adsorbed on aluminum hydroxide adjuvant in vaccine formulation

Linda Cerofolini[‡], Stefano Giuntini[§], Enrico Ravera[§‡], Claudio Luchinat[§‡], Francesco Berti[¥*], Marco Fragai[§‡*]

[‡]. Magnetic Resonance Center (CERM), University of Florence and Consorzio Interuniversitario Risonanze Magnetiche di Metallo Proteine (CIRMMP), Via L. Sacconi 6, 50019 Sesto Fiorentino, Italy

[§]. Department of Chemistry, University of Florence, Via della Lastruccia 3, 50019 Sesto Fiorentino, Italy

[¥]. GSK Vaccines, Technical R&D, Via Fiorentina 1, 53100 Siena, Italy

Supplementary Information

Supplementary Table 1. $^{13}C\alpha$ and ^{15}N chemical shifts of ANSII-AlumOH. The 2D ^{15}N-^{13}C NCA spectrum was collected at 800 MHz (^{1}H Larmor frequency), at ~ 290 K and MAS of 14 kHz.

		Cα	N
P	24	59.48	133.36
N	25	49.31	123.03
I	26	53.02	124.27
I	28	56.42	128.54
L	29	50.60	129.62
A	30	48.05	125.37
T	34	59.43	114.68
I	35	57.07	121.13
L	57	55.10	122.36
V	58	60.96	112.90
N	59	53.75	117.15
A	60	51.83	120.47
V	61	55.03	117.63
P	62	61.77	136.38
Q	63	56.70	119.82
L	64	55.46	118.05
K	65	54.94	113.48
D	66	52.45	114.67
I	67	57.36	112.95
A	68	48.45	120.25
N	69	49.25	118.45
V	70	58.48	125.01
K	71	51.75	127.49
G	72	42.35	111.79
E	73	51.75	120.68
Q	74	52.53	127.01
V	75	62.11	128.78
V	76	56.12	110.28
N	77	49.35	121.59
I	78	61.00	120.26
Q	81	54.91	129.14
D	82	51.03	119.89
M	83	51.66	119.90
N	84	49.02	114.30
D	85	53.30	118.34
N	86	53.77	116.03
V	87	63.38	122.88
W	88	56.05	121.80
L	89	55.62	118.01
T	90	64.25	115.60
L	91	52.10	120.54
A	92	51.19	125.48
K	93	56.65	114.54
K	94	55.34	122.45
I	95	54.97	127.96
N	96	52.52	115.84
T	97	63.46	116.29
D	98	51.83	120.71
C	99	56.63	122.18

D	100	52.30	113.18
K	101	52.81	117.56
T	102	56.18	115.04
D	103	52.32	117.91
G	104	42.48	109.06
F	105	54.38	117.53
V	106	57.09	121.40
I	107	52.66	126.88
H	109	52.36	129.55
G	110	43.00	111.48
M	114	56.75	126.34
E	115	54.74	113.55
E	116	56.16	123.08
T	117	62.01	116.05
K	126	48.99	125.28
C	127	51.07	118.64
D	128	51.97	127.53
K	129	51.63	120.11
P	130	59.67	131.90
V	131	55.59	116.85
V	132	56.31	128.24
V	134	57.72	120.04
G	135	42.09	115.22
A	136	48.78	122.84
M	137	51.40	125.23
R	138	49.86	125.26
V	155	59.10	124.20
V	156	56.97	126.81
T	157	60.04	109.33
A	159	48.93	123.05
D	160	50.94	122.41
K	161	55.89	129.43
A	162	50.18	120.04
S	163	55.69	114.64
A	164	49.53	121.38
N	165	51.96	113.79
R	166	52.32	115.64
G	167	40.29	108.73
V	168	60.85	118.40
L	169	49.78	129.81
V	170	62.62	119.04
V	171	64.10	121.23
R	180	56.03	120.71
D	181	52.56	120.61
V	182	58.92	117.57
T	183	57.21	121.73
K	184	53.95	129.35
T	185	60.99	118.94
N	186	49.31	126.25
T	187	63.28	117.62
T	188	59.77	109.33
D	189	51.10	125.85
V	190	61.97	123.54
A	191	48.75	127.35
T	192	60.68	111.22
F	193	58.43	124.84

K	194	51.22	122.28
S	195	54.80	126.38
V	196	60.37	112.33
N	197	52.44	118.84
Y	198	55.36	118.53
G	199	42.54	104.63
Y	203	59.63	119.25
N	206	50.55	128.31
G	207	42.89	102.29
K	208	51.31	118.90
I	209	56.06	123.07
D	210	48.89	127.32
Y	211	55.64	125.67
Q	212	55.68	120.31
R	213	52.31	120.35
T	214	56.48	114.78
P	215	60.66	133.96
A	216	50.12	123.85
R	217	52.51	116.05
K	218	54.14	121.60
H	219	51.44	116.97
T	220	60.11	110.01
S	221	57.08	118.93
D	222	51.08	120.94
T	223	54.86	109.57
P	224	59.38	130.43
F	225	53.97	120.07
D	226	49.38	122.81
V	227	56.93	118.80
S	228	59.75	117.75
P	234	60.02	132.97
K	235	53.36	124.12
V	236	57.16	126.35
G	237	43.51	116.95
I	238	56.54	117.18
N	241	51.22	120.48
Y	242	51.95	114.28
A	243	50.32	128.00
N	244	50.05	118.28
A	245	50.53	118.74
L	248	49.45	123.07
P	249	64.20	132.12
A	250	51.95	117.09
K	251	56.88	117.66
A	252	52.32	118.96
L	253	51.52	120.96
V	254	62.96	121.55
D	255	54.01	123.72
A	256	48.88	119.87
G	257	43.04	108.45
Y	258	58.43	121.47
D	259	53.97	121.47
V	262	59.38	124.20
S	263	51.98	120.26
A	264	47.07	133.59
G	265	41.98	111.60

V	266	55.97	110.43
G	267	43.12	111.20
N	268	48.08	130.19
G	269	43.94	105.85
N	270	50.61	123.13
L	271	50.32	118.59
Y	272	56.07	124.17
K	273	57.38	129.16
S	274	58.30	113.62
V	275	63.82	126.64
F	276	57.60	122.13
D	277	54.77	118.35
T	278	64.30	117.03
L	279	54.64	122.47
A	280	52.49	124.22
T	281	64.06	115.89
A	282	51.65	125.61
A	283	51.33	122.51
K	284	55.32	118.27
T	285	58.34	108.74
G	286	42.73	108.07
T	287	61.21	122.41
A	288	48.92	130.37
V	289	58.00	124.68
V	290	55.24	125.88
R	291	53.40	126.26
S	292	50.73	120.08
S	293	53.32	115.22
R	294	52.45	126.96
V	295	58.30	125.33
P	296	59.84	132.04
T	297	57.17	110.35
G	298	40.60	109.84
A	299	47.39	118.97
T	300	60.31	121.50
T	301	57.88	119.00
Q	302	53.34	118.90
D	303	49.62	119.19
A	304	48.87	125.38
E	305	54.59	118.82
V	306	56.55	117.53
D	307	49.31	127.78
D	308	56.72	127.02
A	309	51.62	120.96
K	310	55.28	119.67
Y	311	55.62	112.64
G	312	42.69	107.41
F	313	49.45	115.87
V	314	57.51	120.37
A	315	48.49	131.47
S	316	58.04	116.24
L	319	52.24	120.58
N	320	48.52	125.76
Q	322	55.09	113.75
K	323	56.63	124.63
A	324	51.63	120.57

R	325	53.68	120.13
L	327	51.57	113.27
L	328	54.88	120.42
Q	329	58.16	117.55
L	330	53.61	114.80
A	331	53.27	127.75
L	332	53.39	116.49
T	333	60.64	109.54
Q	334	53.08	118.40
T	335	57.60	116.92
K	336	51.63	121.29
D	337	48.53	125.52
P	338	62.23	130.09
Q	339	56.05	117.32
Q	340	54.96	120.06
I	341	63.59	121.77
Q	342	55.61	121.70
Q	343	56.15	118.41
I	344	63.46	122.03

Supplementary Table 2. Parameters used for the acquisition of ^{13}C-detected SSNMR spectra.

**Experiment**	**2D NCA**	**2D NCO**	**2D DARR**
**Transfer 1**	HN CP	HN CP	HC CP
**field [kHz]**	64(H)50(N)	64(H)50(N)	64(H)50(C)
**shape if applicable**	70-100 ramp on H	70-100 ramp on H	70-100 ramp on H
**time [ms]**	1.1	1.1	1.5
**Transfer 2**	NCA DCP	NCO DCP	mixing
**field [kHz]**	80(H)35(N)21(C)	80(H)21(N)35(C)	
**shape if applicable**	tancn shape on C	tancn shape on C	
**time [ms]**	3.5	5	50

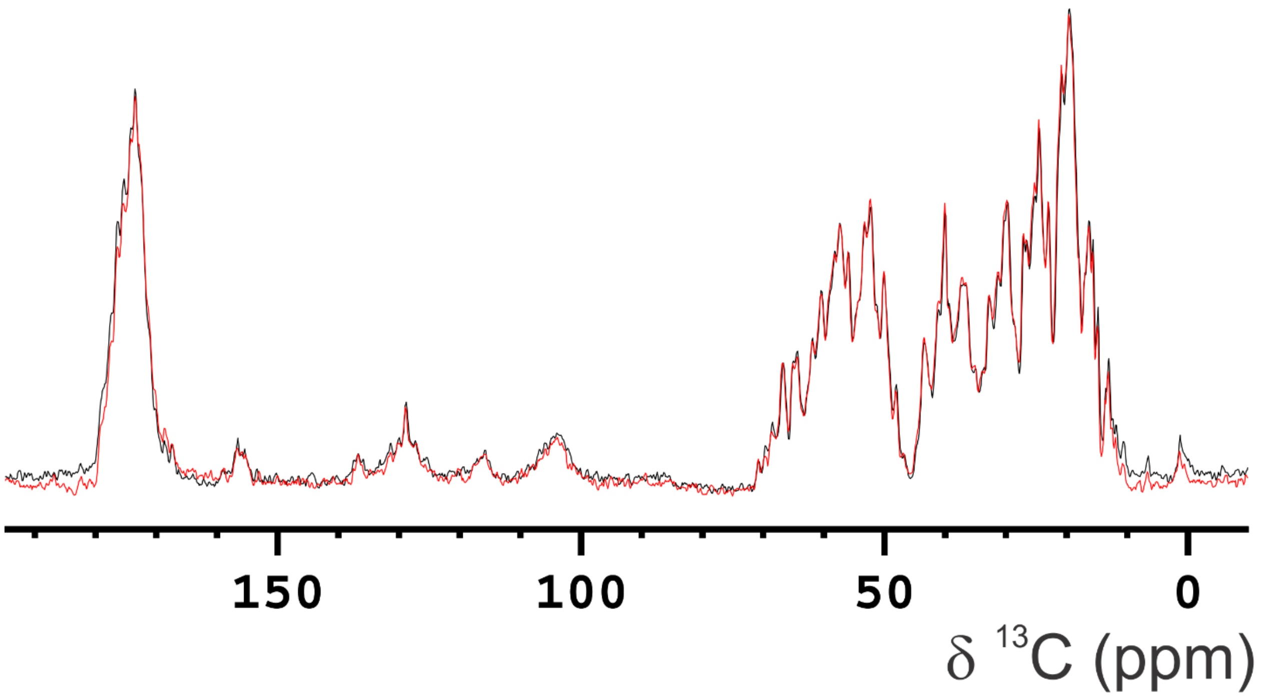

Supplementary Figure 1. 1D {^{1}H}-^{13}C cross-polarization spectra collected just after sample preparation (black) and after whole NMR characterization (red). The spectra were acquired at ~ 290 K, MAS 14 kHz and 800 MHz.
